# Comparative Study of Wheatley’s Trichrome Stain and In-vitro Culture against PCR Assay for the Diagnosis of *Blastocystis* sp. in Stool Samples

**Published:** 2018

**Authors:** Nabilah Amelia MOHAMMAD, Mohd Fahmi MASTUKI, Hesham M. AL-MEKHLAFI, Norhayati MOKTAR, Tengku Shahrul ANUAR

**Affiliations:** 1. Center of Medical Laboratory Technology, Faculty of Health Sciences, Universiti Teknologi MARA, Puncak Alam Campus, Selangor, Malaysia; 2. Endemic and Tropical Diseases Unit, Medical Research Center, Jazan University, Jazan, Kingdom of Saudi Arabia; 3. Dept. of Parasitology, Faculty of Medicine and Health Sciences, Sana’a University, Sana’a, Yemen; 4. Dept. of Pre-Clinical Sciences, Faculty of Medicine and Health Sciences, Universiti Tunku Abdul Rahman, Sungai Long Campus, Selangor, Malaysia; 5. Integrative Pharmacogenomics Institute, Universiti Teknologi MARA, Puncak Alam Campus, Selangor, Malaysia

**Keywords:** Trichrome stain, In-vitro culture, PCR, *Blastocystis*

## Abstract

**Background:**

This study evaluated the performance of routine permanent stain and cultivation method in comparison with polymerase chain reaction assay as the reference technique to detect *Blastocystis* sp.

**Methods:**

A cross-sectional study was conducted among aboriginal populations that reside in Pahang, Peninsular Malaysia in Feb to Mar 2015. A total of 359 stool samples were examined using Wheatley’s trichrome stain, in-vitro cultivation in Jones’ medium and PCR assay. Positive amplicons were subjected to sequencing and phylogenetic analysis.

**Results:**

Fifty-six (15.6%) samples were detected positive with *Blastocystis* sp. by Wheatley’s trichrome stain and 73 (20.3%) by in-vitro culture, while PCR assay detected 71 (19.8%) positive samples. Detection rate of *Blastocystis* sp. was highest in combination of microscopic techniques (27.9%). The sensitivity and specificity of Wheatley’s trichrome staining and in-vitro culture techniques compared to PCR assay were 49.3% (95% CI: 37.2–61.4) and 92.7% (95% CI: 89.1–95.4) and 39.4% (95% CI: 28.0–51.8) and 84.4% (95% CI: 79.7–88.4), respectively. However, the sensitivity [60.6% (95% CI: 48.3–71.9)] of the method increased when both microscopic techniques were performed together. False negative results produced by microscopic techniques were associated with subtype 3. The agreement between Wheatley’s trichrome stain, in-vitro culture and combination of microscopic techniques with PCR assay were statistically significant by Kappa statistics (Wheatley’s trichrome stain: *K* = 0.456, *P*<0.001; in-vitro culture: *K* = 0.236, *P*<0.001 and combination techniques: *K* = 0.353, *P*<0.001).

**Conclusion:**

The combination of microscopic technique is highly recommended to be used as a screening method for the diagnosis of *Blastocystis* infection either for clinical or epidemiological study to ensure better and accurate diagnosis.

## Introduction

*Blastocystis* sp. is a unicellular enigmatic protozoan parasite commonly isolated from humans and animals stool samples with unsettled clinical significance. It possesses higher prevalence ranging from 1% to 60% in developed and developing countries (1–3). Blastocystosis is an infection caused by *Blastocystis* sp. characterized by symptoms such as diarrhea, abdominal pain, nausea, loss of weight and vomiting (4, 5). This parasite was suggested to be transmitted through fecal-oral route via contamination of food and water in both humans and animals (1, 6). *Blastocystis* sp. (STs) showed an extensive genetic diversity with currently recorded 17 distinct subtypes based on the analysis of the small subunit ribosomal RNA (SSU rRNA) gene, comprising different hosts; the humans and wide array of animals (7). ST1-ST9 are found in humans with some isolates from humans has been demonstrated to be closely related to animals isolates. ST10-ST17 is exclusively found in animals. Majority of human infections are attributed to ST3 (7, 8).

Detection of *Blastocystis* sp. largely depends on the microscopic examination with wet mount preparation or permanent stain. Being cost effective and time-consuming are the reasons why these methods still widely employed in the diagnostic laboratories (9, 10). Microscopic examination relies on the detection of four different morphologies of *Blastocystis* sp. including vacuolar, granular, cysts and amoeboid forms. However, this diagnostic method usually leads to misidentification as polymorphic morphologies of *Blastocystis* sp. possess great variation in term of size and shape (10, 11). In-vitro cultivation of viable *Blastocystis* sp. with non-standardized microorganisms had been widely addressed as the gold standard for its detection. Its usefulness has been demonstrated by numbers of researchers worldwide (12, 13). It is a huge advantage when observing samples from symptomatic subjects. Amoeboid form that commonly found among these subjects tends to be bigger in size thus, can be easily detected. However, the inabilities of the technician to recognize this form often lead to underreported (14).

Molecular approaches involving polymerase chain reaction (PCR) assay has become method of choice for diagnosing *Blastocystis* infection. It is astounding ability in revealing the genetic information of the parasite helps in providing further knowledge on the subtype's distribution among different hosts, its zoonotic risks, and pathogenicity of different subtypes (15, 16). However, most of the developing countries cannot afford to use PCR as a part of their diagnostic tool because it required fancy tools that are complex and expensive. It also requires specialized and highly trained technician to promote intended results (17). Hence, microscopic examination consisted permanent stain and in-vitro culture are still favorable in detecting *Blastocystis* sp.

The main objective of this study was to evaluate the sensitivity, specificity, and agreement of two methods (Wheatley’s trichrome stain and in-vitro culture) in detection of *Blastocystis* sp. in stool samples compared with PCR assay as a reference technique.

## Materials and Methods

A cross-sectional study was conducted among aboriginal populations that reside in Pahang, Peninsular Malaysia. Three-hundred and fifty-nine stool samples were collected from different hosts over the period of two months (Feb to Mar 2015). Of it, 253 samples were collected from the aboriginal. The remaining 106 samples were collected from domestic animals and livestock such as chickens, birds, dogs, cows, ducks, macaque, cat, geese, rabbits, sheep and deer that were reared and breed by these populations and Sungai Jin Deer Farm that located within the aboriginal settlements.

Informed consent was taken from the participants and the study was approved by the Ethics committee of the university.

Samples were processed at the designated work area located adjacent to the study site within four hours after samples collection. Samples were divided into three portions; (i) 5 g of stool were fixed in 15 ml centrifuge tube containing 3 ml polyvinyl alcohol (PVA), stored at room temperature for later Wheatley’s trichrome stain; (ii) approximately 10 mg of stool were transferred into new stool container, stored at room temperature for in-vitro culture; (iii) the remaining stool were kept in the original stool container and stored at −20 °C for genomic DNA extraction and PCR analysis. All stool samples were processed within 2 to 4 wk after collection.

### Wheatley’s trichrome stain

**Fig. 1:**
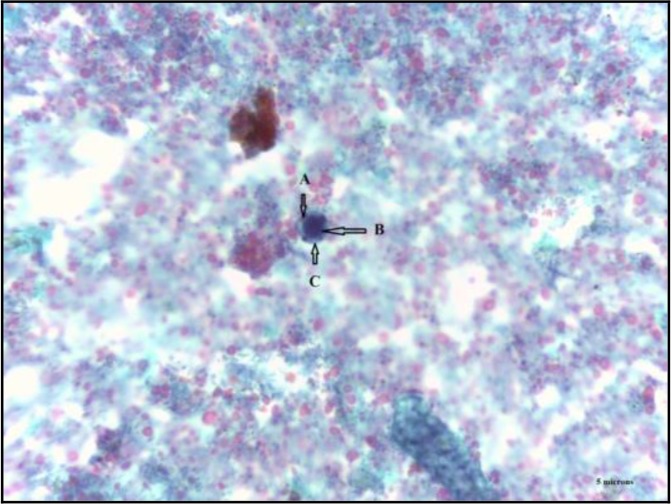
Vacuolar form with four nucleuses found at the rim of cytoplasm along with large and round vacuole at the center. a) Nucleus. b) Vacuole. c) Cytoplasm

Samples fixed in PVA were filtered, centrifuged and dried prior to permanent staining. The smeared cover slips were allowed to air-dry and stained with Wheatley’s trichrome stain (18). Slides were mounted with Distrene, Plasticiser, and Xylene (DPX) (Sigma Aldrich) and observed microscopically under 100x magnification for the presence of *Blastocystis* sp. Slides were cross-checked by experienced technologist. A sample was reported as positive if any of the four morphologies (vacuolar, granular, cyst and amoeboid) of *Blastocystis* sp. were seen (Fig. 1).

### In-vitro culture

**Fig. 2:**
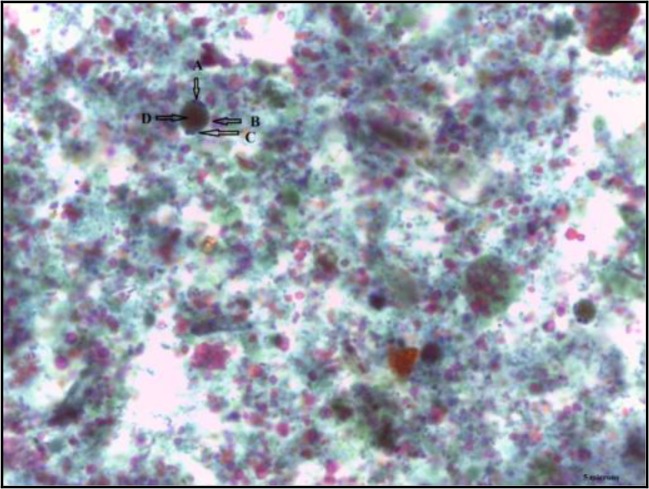
Granular form revealed large central vacuole filled with dense granules and nucleus located along the thin rim of cytoplasm. a) Nucleus. b) Vacuole. c) Cytoplasm. d) Granules

Inoculation of 5 mg stool samples was carried out into 5 ml screw-capped tubes containing 3 ml Jones’ medium using sterile applicator stick (10, 19). The culture medium was supplemented with 10% heat-inactivated horse serum (Gibco, USA) prior to use. The culture tubes were incubated at 37 °C. The presence of *Blastocystis* sp. was observed daily for 3 d (humans) and 7 d (animals) of cultivation by placing one drop of culture products onto PVA-coated coverslips and stained with Wheatley’s trichrome. Prepared slides were examined microscopically at a magnification of 100x and cross-checked by senior technologist. Sample was reported as positive if any of the four morphologies (vacuolar, granular, cyst and amoeboid) of *Blastocystis* sp. were observed (Fig. 2).

### Genomic DNA extraction and molecular analysis

Genomic DNA was directly extracted from all unfixed stool samples using QIAamp^®^ Fast DNA Stool Mini Kit (QIAGEN, Hilden, Germany) according to the manufacturer’s instructions. The final DNA elution was made in 100 μl of elution buffer and stored at −20 °C until required for PCR amplification.

The conventional PCR was conducted on the extracted DNA using primer F1: 5′-GGA GGT AGT GAC AAT AAA TC-3′ (20) and BHCRseq3: 5′-TAA GAC TAC GAG GGT ATC TA-3′ (21) which targeted the SSU rRNA of *Blastocystis* sp. PCR amplification was performed in a final volume of 50 μL per reaction containing 25 μL TopTaq Master Mix kit (QIAGEN, Hilden, Germany), 17 μL nuclease-free water, 4 μL template DNA and 2 μL of each primer. Amplification was carried out in Nyx Technik thermal cycler (Nyx Technik, USA) and the following amplification profile was used: 3 min of initial denaturation at 94 °C, followed by 35 cycles of denaturation at 94 °C for 30 sec, annealing at 59 °C for 30 sec and extension at 72 °C for 1 min. Final extension was conducted at 72 °C for 10 min. Fragment of 550 to 585 basepairs of the amplified PCR products were separated on a 1.5% (w/v) agarose gel (Vivantis Technologies, USA) at 70 V for 110 min (Bio-Rad Laboratories, USA), stained with GelRed (Biotium, USA), visualized under ultraviolet light and photographed using Sastec ST GD 1510 (SASTEC^™^ Instrument Inc., Canada). Positive and negative controls were included in every PCR reactions. *Blastocystis* ST1, ST2, and ST3 were used as positive control, whereas, nuclease-free water was used as negative control replacing template DNA.

Sequencing was performed on all PCR positive samples. Sequences obtained in the present study were aligned and edited manually with reference sequences of *Blastocystis* sp. (ST1-ST14) using ClustalX2.1 software. Phylogenetic tree was constructed using maximum likelihood method (1000 bootstrap replicates) based on Hasegawa. Kishino. Yano+G (Gamma distribution)+ I(Invariant) model (best-fit substitution model) using MEGA 6.06 software (22). *Proteromonas lacertae* (U37108) was used as outgroup.

### Statistical analysis

Statistical analysis of the data was performed using the SPSS software for Windows Ver. 20 (Inc. Chicago, IL, USA). Calculation involving sensitivity, specificity, positive predictive value (PPV) and negative predictive value (NPV) with exact binomial 95% confidence intervals (95% CI) were used to assess the applied methods. The weighted Kappa statistic was used to assess the agreement between the methods used. The assessment of agreement was as follows: >0.81 indicate perfect agreement, 0.61–0.80 substantial agreement, 0.41–0.60 moderate agreement, 0.21–0.40 fair agreement, 0.01–0.20 slight agreement and <0.20 poor agreement (23). A *P*-value <0.05 was indicated as the level of significance.

## Results

**Fig. 3:**
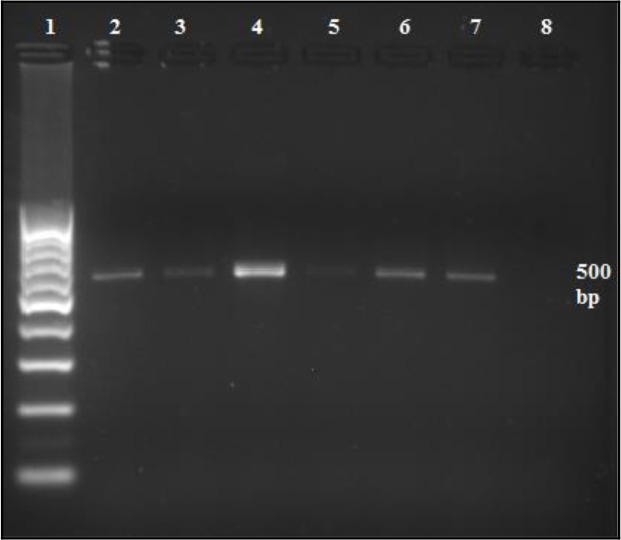
Analysis of PCR products on a 1.5% (w/v) agarose gel. Lane 1) 100-bp DNA ladder, Lane 2) Negative control, Lane 3) Positive control, Lane 4–8) Positive *Blastocystis* isolates

**Fig. 4:**
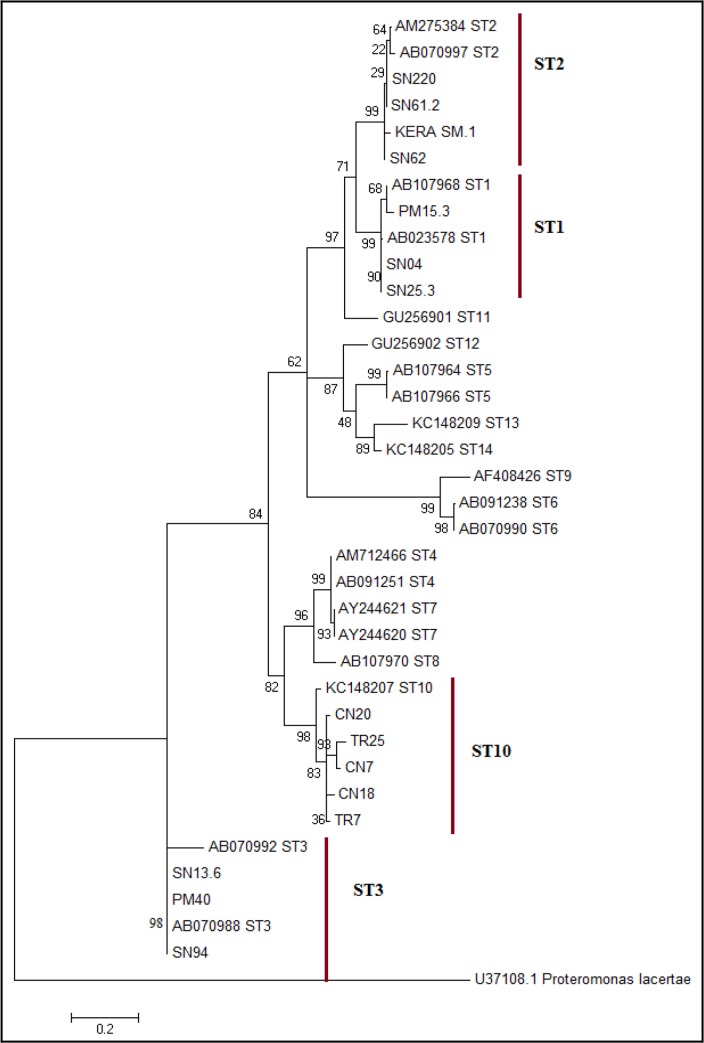
Maximum likelihood tree displaying relationship amongst SSU rRNA gene sequences of *Blastocystis* from this study (bold line) with reference sequences (ST1-ST14) from GenBank. *P. lacertae* served as outgroup

The prevalence rates of *Blastocystis* sp. in all examined stool samples were 15.6% (Wheatley’s trichrome stain), 20.3% (in-vitro culture) and 16.2% (PCR assay). Of 71 samples that were positive by PCR (Fig. 3), only 58 readable sequences were obtained. Fifty-eight isolates could be separated into 4 subtypes contained isolates from humans and animals (Fig. 4).

Among human isolates, ST3 (9.5%) was the predominant subtype, followed by ST1 (5.5%) and ST2 (2.8%). Results of animal positive isolates showed that ST10 was predominant among deer (11.3%), while ST2 (1.0%) was found in macaque.

Phylogenetic analysis showed higher detection rate was observed when both microscopic methods were performed together (27.9%) as compared to individual method (Table 1). PCR assay managed to amplify SSU rRNA gene sequence of *Blastocystis* in 28 samples initially observed as microscopy-negative. However, PCR assay was unable to amplify 50 (13.9%) stool samples initially diagnosed as positive by both methods.

**Table 1:** Comparison of different diagnostic methods in detection of *Blastocystis* sp.

***Diagnostic tests***	***Results***	
	Positive n (%)	Negative n (%)
Wheatley’s trichrome stain	56 (15.6)	303 (84.4)
In-vitro culture	73 (20.3)	286 (79.7)
Combination techniques	100 (27.9)	259 (72.1)
PCR assay	71 (19.8)	288 (80.2)

The performance of Wheatley’s trichrome stain and in-vitro culture in term of sensitivity, specificity, PPV, and NPV in comparison to PCR assay (reference technique) were presented in Table 2.

**Table 2:** Sensitivity, specificity, PPV, and NPV of two microscopic diagnostic methods compared to PCR assay

	***Wheatley’s trichrome stain***		***In-vitro culture***	
	+ve	−ve	+ve	−ve
+ve (n = 71)	35	36	28	43
PCR				
−ve (n = 288)	21	267	45	243
Total	56	303	73	286
Sensitivity (%) (95% CI)	49.3 (37.2–61.4)		39.4 (28.0–51.8)	
Specificity (%) (95% CI)	92.7 (89.1–95.4)		84.4 (79.7–88.4)	
PPV (%)	62.5		38.4	
NPV (%)	88.1		85.0	
Kappa (*P*-value)	*K* = 0.456 (*P*<0.001)*^a^*		*K* = 0.236 (*P*<0.001)*^a^*	

CI; confidence interval.

PPV; positive predictive value.

NPV; negative predictive value.

a ; significant association (*P*<0.05)

The Wheatley’s trichrome stain showed higher sensitivity [49.3% (95% CI: 37.2–61.4)] and specificity [92.7% (95% CI: 89.1–95.4)] compared to in-vitro culture technique, which only showed 39.4% sensitivity (95% CI: 28.0–51.8) and 84.4% specificity (95% CI: 79.7–88.4).

However, when both diagnostic methods were performed together, the results showed high sensitivity [60.6% (95% CI: 48.3–71.9)] but the specificity decreased to 80.2% (95% CI: 75.1–84.7) (Table 3). The agreement between the PCR assay and Wheatley’s trichrome stain was statistically significant by Kappa (*K*=0.456; *P*<0.001). Similar results were also obtained between PCR assay and in-vitro culture (*K* = 0.236; *P*<0.001) and PCR assay and combination of microscopic techniques (*K* = 0.353; *P*<0.001).

**Table 3:** Sensitivity, specificity, PPV, and NPV of combination microscopic techniques compared to PCR assay

	***Combination techniques***	
	+ve	−ve
+ve (n = 71)	43	28
PCR		
−ve (n = 288)	57	231
Total	100	259
Sensitivity (%) (95% CI)	60.6 (48.3–71.9)	
Specificity (%) (95% CI)	80.2 (75.1–84.7)	
PPV (%)	43.0	
NPV (%)	89.2	
Kappa (*P*-value)	*K* = 0.353 (*P*<0.001)*^a^*	

CI; confidence interval.

PPV; positive predictive value.

NPV; negative predictive value.

a; significant association (*P*<0.05)

## Discussion

Microscopic examination using wet mount, concentration technique and variety of permanent stain have been widely employed to identify *Blastocystis* sp. Easy access to the resources and less time required to conduct these methods are the factors contributing to its frequent adoption as the routine diagnostic methods (17, 24). However, morphology-based diagnosis usually goes underreported as the vegetative stages of *Blastocystis* sp. often mistaken for fat globules and other contaminants (11). In this study, Wheatley’s trichrome stain showed high sensitivity (49.3%) and specificity (92.7%) despite detecting slightly lower prevalence rate (15.6%). Wheatley’s trichrome stain was able to correctly identify those subjects having blastocystosis. The uses of preservatives prior to staining procedure retain the morphology of protozoan parasites thus increasing the detection rate (25, 26). A very strong agreement of trichrome stain when culture was used as the reference technique (*K*=0.922) (27). In concordance to that, 100% specificity of Wheatley’s trichrome stain in detecting *Blastocystis* sp. from irritable bowel syndrome/irritable bowel disease patients proved it diagnostic accuracies (28). Wintergreen oil was used to replace xylene as the clearing agent. The usage of wintergreen oil gave a high clarity of the image observed as it deeply stained the cytoplasm, peripheral chromatin, karyosome and provide better background that further ease the observation (18).

In-vitro culture gave higher prevalence than Wheatley’s trichrome stain which was 20.3%. However, when testing the performance and effectiveness of in-vitro culture against the reference technique, in-vitro culture resulted in the highest detection of false positive which was in 45 samples. This corresponds to lower sensitivity value of 39.4%. This finding was supported by previous studies which reported that in-vitro culture showed no significant differences with comparable detection accuracies as compared to trichrome stain. This showed that in-vitro culture does not necessarily superior and sensitive than other diagnostic methods (13, 29). In-vitro culture unable to detect *Blastocystis* sp., although some of the samples were positive by PCR technique (30). In-vitro culture was rather insensitive despite being a renowned gold standard for *Blastocystis* sp. detection (16). Apparently, there are a lot of culture media available such as Jones’ medium and Iscove’s modified Dulbecco’s medium with each of it possess different reagents, composition, and protocols in order to grow the viable *Blastocystis* sp. These differences contribute to a variation of sensitivity and specificity of culture technique recorded worldwide (30, 31). An optimization and standardization of various culture techniques are urgently needed in order to curb with the ineffectiveness and underperformance of this technique. Furthermore, viable *Blastocystis* sp. can easily degenerate and intermittent shedding regularly occur in the fresh stool samples, hence, lower the chances of the parasite to grows and detected during cultivation (21, 32).

The present study showed that when both microscopic techniques (Wheatley’s trichrome stain and in-vitro culture) were used together, the overall prevalence and sensitivity of the *Blastocystis* sp. detection was markedly improved and increased, which were 27.9% and 60.6%, respectively. It concurs with postulation made, at least two diagnostic methods should be used together to ensure accurate diagnosis whenever molecular technique cannot be adopted (16). Being solely depends on one technique could lead to underreported results. Wheatley’s trichrome stain alone has proved to successfully detect the presence of *Blastocystis* sp. in stool samples by giving color to the cytoplasm (blue-green to purple) and nuclei (red or purple-red), thus ease the detection (18). However, this microscopy-based technique was greatly affected by the number of parasite presence in the stool samples. Hence, the employment of in-vitro is equally important as it will allow *Blastocystis* sp. to grow. This method will increase the number and size of the parasite and makes the identification through light microscopy much easier (31, 33).

Nowadays, detection of *Blastocystis* through PCR-based method has become the most favorable diagnostic method. PCR assay was widely used as reference technique because of its high sensitivity and specificity over other microscopy methods, as well as its ability to reveal the genetic information of *Blastocystis* (15, 30). This corresponded to the detection of four subtypes in this study which was ST1, ST2, ST3, and ST10 that constitute both human and animal samples. The predominant of ST3 among human isolates were in concordance with other studies conducted worldwide (34, 35). Furthermore, through molecular analysis, the ability of Wheatley trichrome stain and in-vitro culture to detect *Blastocystis* sp. depends on the subtypes. False negative result appeared to be associated with *Blastocystis* subtype 3. ST3 of *Blastocystis* might encyst before shedding; hence the present of inconspicuous cyst was hardly recognized by these microscopy techniques (21). PCR assay also presented with many disadvantages. Unsuccessful DNA extraction and the choice of unsuitable primers could lead to no and/or over-amplification of PCR assay (12). Furthermore, PCR is a complex and costly method to be adopted by developing countries as advanced equipment and specialized personnel are needed to run this technique.

## Conclusion

We suggest the use of combination of Wheatley’s trichrome stain and in-vitro culture as screening tools for detection of *Blastocystis* sp. especially in community laboratories that facing financial constraints and are not equipped with molecular facilities. The high specificity shown by both microscopic techniques indicates that it can be used to identify those not infected with *Blastocystis* sp. with high diagnostic accuracies. However, whenever applicable, PCR assay should acts as confirmatory test as it’s the only method that can recognize subtypes of *Blastocystis.*
